# Complete mitochondrial sequencing reveals the horizontal transfer and gene rearrangement of *Passiflora edulis*

**DOI:** 10.3389/fpls.2026.1851081

**Published:** 2026-05-28

**Authors:** Yao Teng, Ziyuan Hao, Yuhan Liu, Caixia Chen, Sunjian Zhang, Ye Wang, Yan Sun, Hongyuan Jing, Xiuqin Long

**Affiliations:** 1 Guizhou Institute of Mountain Resources, Guizhou Academy of Sciences, Guiyang, Guizhou, China; 2 Pingtang Karst Peak-cluster Depression Ecosystem Observation and Research Station of Guizhou Province, Pingtang, Guizhou, China; 3 College of Horticulture and Plant Protection, Henan University of Science and Technology, Luoyang, Henan, China; 4 Guizhou Botanical Garden, Guizhou Academy of Sciences, Guiyang, Guizhou, China

**Keywords:** genomic rearrangement, hybrid sequencing, mitochondrial DNA, mitochondrial plastid DNA (MTPTs), passion fruit

## Abstract

**Introduction:**

The mitochondrial genome (mitogenome) of *Passiflora edulis*, an economically important fruit crop, remains poorly characterized, with previous reports suggesting a simple circular structure.

**Methods:**

We employed a hybrid sequencing strategy combining PacBio HiFi long reads and Illumina short reads to assemble the complete mitogenome of *P. edulis* variety 'GH-1'. The assembly was validated by PCR and Sanger sequencing. We further analyzed repeat sequences, transposable elements, mitochondrial plastid DNA (MTPT), codon usage, and performed comparative genomic and phylogenetic analyses.

**Results:**

The *P. edulis* mitogenome comprises two circular chromosomes (mtChr1: 380,183 bp; mtChr2: 334,822 bp), a novel double-circular conformation. It encodes 50 functional genes, 21 tRNAs, and harbors 59 SSRs and 56 long forward repeats. A total of 1,596 transposable elements (predominantly LTR retrotransposons) and nine MTPT fragments (totaling 43,909 bp, 6.14% of the mitogenome) were identified. Comparative analysis revealed extensive gene rearrangements between the purple (PES) and yellow (PEF) varieties.

**Discussion:**

The double-circular conformation is likely mediated by abundant forward repeats. The high proportion of MTPTs highlights the dynamic nature of inter-organellar gene transfer. This high-quality mitogenome provides critical insights for phylogenetic studies and breeding programs in Passifloraceae.

## 1 Introduction

Mitochondria are fundamental organelles in plants which originating from an ancient endosymbiotic event. They serve as primary producers of ATP and play critical roles in cellular signaling, growth regulation, and stress responses (Ogihara et al., 2005). As semi-autonomous organelles, mitochondrial genomes evolve in markedly different ways from the nuclear genome. Despite their importance, plant mitochondrial DNAs (mtDNAs, or mitogenomes) have received less attention than the nuclear and chloroplast counterparts, partially because their complex architecture has long hindered comprehensive characterization. In plants, mtDNA is uniparentally inherited and exhibits remarkable diversity in size, structural conformation, and gene arrangement (Chen and Butow, 2005; Nosek and Tomaska, 2003). The traditional view that mtDNA exists as a single, circular conformation has been increasingly challenged. Emerging evidence indicates that this depiction is often an oversimplification. Owing to frequent recombination events mediated by repetitive sequences, plant mitogenomes frequently adopt intricate multi-chromosomal configurations, including multiple circular, linear, or branched DNA molecules (Gualberto and Newton, 2017; Sloan et al., 2012a). In contrast, the chloroplast genome (cpDNA) of most angiosperms is relatively conserved in structure, gene order, and uniparental (typically maternal) inheritance (Palmer et al., 1988; Whatley, 1982).

The size of plant mitogenomes is highly variable, ranging from approximately 100 kbp in the bryophyte *Mielichhoferia elongata* to 11.3 Mbp in *Silene conica* (Goryunov et al., 2018). Even within the genus *Silene*, size spans from 253 kbp to 11 Mbp, differences largely attributable to the expansion or contraction of intergenic regions (Sloan et al., 2012b). This extensive variation in size, structure and gene content is frequently shaped by recombination, horizontal gene transfer (HGT), and the integration of foreign DNA (Hepburn et al., 2012). Recent studies has revealed that plant mtDNA can incorporate sequences from chloroplast (MTPTs), leading to genomic mosaicism and challenging the traditional view of strict vertical inheritance (Wang et al., 2007; Wu et al., 2022). Such dynamic intergenomic exchanges not reshape organellar architecture but have implications for phylogenetic reconstruction and our understanding of cytoplasmic inheritance.

The complexity of plant mitogenomes is further exacerbated by rampant inter-organellar DNA transfer. Frequent HGT occurs between mitochondria and plastids, as well as between mitochondria and the nucleus, blurring the boundaries between genomic compartments (Filip and Skuza, 2021). These phenomena have long made the chloroplast genome a more tractable target for plant genomic studies, while the mitochondrial counterpart remains largely unexplored in most plant species. Until recently, the vast majority of angiosperm mitogenomes remained uncharacterized. The situation has changed dramatically with the advent of long-read sequencing technologies, such as Oxford Nanopore and PacBio HiFi. These platforms enable the resolution of complex structural variations and repetitive regions that were previously intractable with short-read sequencing alone. In *Abelmoschus esculentus*, a combination of Illumina short reads and nanopore reads allowed the assembly of near-complete, circular mitogenomes, subsequently used to refine phylogenetic relationships (Li et al., 2022). A hybrid long- and short-read strategy of *Ventilago leiocarpa* revealed a complex branched mitochondrial architecture that had escaped detection in earlier assemblies (Guo et al., 2024). Collectively, these cases underscore how contemporary sequencing methodologies have elevated assembly quality, unveiling the true, often intricate, structural nature of plant mitogenomes and opening new avenues for comparative genomics.

The genus *Passiflora* (commonly known as passion fruit) belongs to the family Passifloraceae and is of considerable economic importance. It comprises over 500 species distributed across the world (Cerqueira-Silva et al., 2014). Among these, Approximately 95% of these accessions are located in germplasm banks of Brazil, Ecuador, Peru, Colombia, France and USA (Cerqueira-Silva et al., 2015). Based on the pericarp coloration of passion fruit, cultivars were divided into purple passion fruit (PES, *P. edulis Sims*) and yellow passion fruit (PEF, *P. edulis* f. *flavicarpa*) (Bernacci et al., 2008; Castillo et al., 2020). PEF cultivar is orange–yellow and sweet, while PES cultivar is deeper orange and less sweet than PEF (Teng et al., 2024). Driven by their unique aromatic flavor and high antioxidant content, both purple and yellow passion fruit cultivars have gained considerable commercial interest, stimulating the generation of genomic resources for *Passiflora*. The chloroplast genome of *P. edulis* was among the first organellar genomes assembled for the genus, revealing the canonical quadripartite structure and providing a robust phylogenetic framework for Malpighiales (Cauz-Santos et al., 2017). Recently, high-quality chromosome-level nuclear genome assemblies have become available for *P. edulis*, identifying key gene families associated with flavor biosynthesis, disease resistance, and environmental adaptation (Xia et al., 2021; Zheng et al., 2024). These nuclear and plastid references have accelerated molecular breeding and phylogenetic inference within Passifloraceae. However, only a draft mitochondrial genome assembly of PEF based on short reads, which reported a single-circular conformation (Yang and Wang, 2020). as we will demonstrate, that draft substantially underestimates the true complexity of the *Passiflora* mitogenome. *Passiflora* occupies a key phylogenetic position within *Malpighiales*, a rosid order for which mitogenomic architecture remains poorly documented. The presence of extensive repetitive sequences and MTPTs in this genome offers a unique opportunity to examine how non-nuclear genomes diversify in a lineage that has undergone rapid speciation. Also, Mitochondrial sequence evolve distinct selective constraints compared to chloroplast spacers, hold promise as high-resolution markers for resolving species boundaries and authenticating commercial cultivars in a genus where vegetative morphology is often ambiguous.

In this study, we present the complete mitochondrial genome sequence of the PES representative variety ‘GH-1’, assembled using a hybrid strategy that combines PacBio HiFi long reads and Illumina short reads. Our specific objectives are: (1) to characterize the fundamental features, particular attention to the conformation of *P. edulis Sims*; (2) to analyze the distribution patterns of repetitive sequences and transposable elements and assess genomic rearrangements between PES and draft PEF fruit; (3) to quantify the extent of mitochondrial-plastid DNA transfer (MTPT) and to explore its potential role in shaping the mitogenome. By integrating comparative genomic and phylogenomic approaches, this work aims to elucidate the extent of mitochondrial-plastid DNA transfer and rearrangement and clarify the mechanisms underlying mitogenome instability in Passiflora.

## 2 Materials and methods

### 2.1 Plant material

The cold-tolerant passion fruit variety ‘GH- 1’ (registration number: Qianrenguo20220006, Guizhou, China) was used as the experimental material. This variety was newly bred by our research group in 2023 (Teng et al., 2024). Fresh leaves of *P. edulis* were collected from the Guizhou Academy of Sciences, Guiyang, China, on April 20, 2025. Leaf samples were excised, immediately frozen in liquid nitrogen, and stored at -80 °C until DNA extraction. Total genomic DNA was extracted using the CTAB method following the manufacturer’s protocol (DP441, TIANGEN). DNA concentration was initially quantified using a NanoDrop ONE instrument, with acceptable absorbance ratios of 260/280 between 1.8 and 2.0 and 260/230 greater than 2.0. Precise quantification was then performed using the Qubit fluorometric method, requiring a Nc/Qc ratio of 0.95–1.5. The final DNA concentration of each sample was ensured to be no less than 500 ng/μL. DNA integrity was assessed by agarose gel electrophoresis and confirmed with an Agilent 2100 Bioanalyzer, requiring an RIN value greater than 7.

### 2.2 Hybrid sequencing library preparation

After quality control, the DNA sample was divided into two aliquots. One was used to construct a library for long-read sequencing on the PacBio Revio platform (TGS, third-generation sequencing), and the other was used for short-read sequencing (NGS, next-generation sequencing) on the BGI DNB-seq T7 platform (Frasergen, Wuhan, China). For TGS, approximately ~ 2 μg of genomic DNA was used to construct SMRTbell libraries with the PacBio Express Template Prep Kit 2.0. The genomic DNA was fragmented, and fragments of desired size were selected using BluePippin. After end-repair and A-tailing, adapters were ligated to both ends of the fragments to prepare the DNA library. For NGS, approximately ~ 2 μg of DNA was fragmented into ~350 bp fragments to construct paired-end libraries. The sonicated DNA was purified using a TIANgel Midi Purification Kit, and adapters were ligated using the Nextera DNA Sample Preparation kit. Paired-end reads of 2 × 150 bp were sequenced on the DNB-seq T7 platform according to the manufacturer’s instructions. After sequencing, the short reads were subjected to quality control using fastp software (Chen et al., 2018).

### 2.3 Assembly and validation of mitochondrial and chloroplast genomes

The TGS reads were filtered using hififilter software (Sim et al., 2022) and then aligned to the complete mitochondrial genomes of *Populus* (Qu et al., 2023), *Arabidopsis thaliana* (Park et al., 2021), and the draft mitochondrial genome of *P. edulis* (Yang and Wang, 2020) retrieved from NCBI using minimap2 software (Li, 2018). Long-reads with an alignment length greater than 5,000 bp were extracted for subsequent assembly. The NGS reads were filtered with fastp software (Chen et al., 2018). After removing adapters, unpaired reads, short reads, and low-quality reads, the remaining high-quality reads were aligned to the entire mitochondrial genome using bowtie2, and the successfully aligned reads were retained for polishing and refining the TGS assembly.

The complete mitochondrial genome assembly was performed in four main steps: ① *de novo* assembly of the filtered long reads using oatk software (Zhou et al., 2025) to obtain an initial assembly graph; ② *de novo* assembly of all long reads using the newly developed himt software to generate an updated assembly graph (Tang et al., 2025); ③ optimization of the two assembly graphs from different software based on himt compare and synteny analysis, resulting in a relatively complete mitogenome map (Tang et al., 2025); ④ refinement of the assembled mitogenome sequence map using NGS short reads (Walker et al., 2014). To further verify the accuracy and completeness of the mitogenome, gene-specific primers were designed to amplify and validate the junction regions between different contigs in the assembly (primer sequences are listed in **Supplementary Table 1**). Based on the validation results, the structure and sequence of the complete ‘GH-1’ mitogenome were confirmed.

Finally, using the reference genomes of *Arabidopsis thaliana*, *Populus*, and the previously reported draft mitogenome of *Passiflora*, annotation of the assembled complete ‘GH-1’ genome was performed using the online tool PMGA (http://www.1kmpg.cn/pmga/), and a physical map of the mitogenome was generated (Li et al., 2025). Transfer RNA (tRNA) genes were annotated using the tRNAscan-SE online website (http://lowelab.ucsc.edu/tRNAscan-SE/) (Chan et al., 2021). Ribosomal RNA (rRNA) genes were annotated using the RNAmmer 1.2 Server (http://www.cbs.dtu.dk/services/RNAmmer/) (Lagesen et al., 2007). The final annotation results were obtained after manual correction. Subsequently, OGDRAW (https://chlorobox.mpimp-golm.mpg.de/OGDraw.html) was used to generate a physical genome map (Greiner et al., 2019).

### 2.4 Analysis of repeat sequences and transposable elements

Simple sequence repeat (SSR) loci were analyzed using the online tool MISA (https://webblast.ipk-gatersleben.de/misa/) (Beier et al., 2017). The parameters were set as follows: 10 repeats for mono-, 6 repeats for di-, 5 repeats for tri-, tetra−, and pentanucleotides, and a maximum distance of 100 bp between two SSR loci. REPuter (https://bibiserv.cebitec.uni-bielefeld.de/reputer) was used to detect forward, reverse, complement, and palindromic repeats in the genome, with a minimum repeat length of 30 bp and an E−value threshold of 1e−5 (Kurtz et al., 2001). Transposable elements were identified using CENSOR (https://www.girinst.org/censor/index.php) based on the Gypsy Database (GyDB), allowing determination of transposable element types and their distribution (Llorens et al., 2010).

### 2.5 Identification of mitochondrial plastid DNAs

To identify DNA fragments potentially transferred from the plastome, a BLASTn search was performed using TBtools software (Chen et al., 2023), with the assembled *Passiflora* chloroplast genome sequence as a reference against the mitogenome sequence. The E−value threshold was set to 1e−5. Chloroplast−derived sequences (MTPTs) within the mitogenome were thus identified. The lengths of the identified MTPTs were calculated, functional annotation was performed, and their distribution characteristics within the mitogenome were analyzed.

### 2.6 RNA editing and codon usage bias analysis

RNA editing sites in mitochondrial protein−coding genes were predicted using the RNA editing site prediction tool of the PMGA online platform (http://www.1kmpg.cn/pmga/). The predicted editing sites were classified and statistically analyzed to examine their distribution across different genes and at different codon positions. Codon usage bias was analyzed using CodonW software (Peden, 2000), and the relative synonymous codon usage (RSCU) values were calculated. An RSCU value greater than 1 indicated preferential usage of a given codon. Visualization of the results was performed using local Python scripts.

### 2.7 Phylogenetic analysis

Mitochondrial genome CDS sequences of 16 representative angiosperm species were downloaded from the NCBI database, including species from Salicaceae, Brassicaceae, Fabaceae, Solanaceae, and other families. Orthologous gene pairs among the different species were identified using OrthoFinder software (Emms and Kelly, 2019). Subsequently, multiple sequence alignments of the orthologous genes between Passiflora and the other species were performed using MAFFT software (Katoh and Standley, 2013). The aligned nucleotide sequences were concatenated and used to construct phylogenetic trees using the maximum−likelihood (ML) method implemented in iqtree software (Nguyen et al., 2015). The resulting phylogenetic tree was visualized using FigTree software.

## 3 Results

### 3.1 Assembly and validation of complete chloroplast and mitochondrial genomes

**Figure 1:**
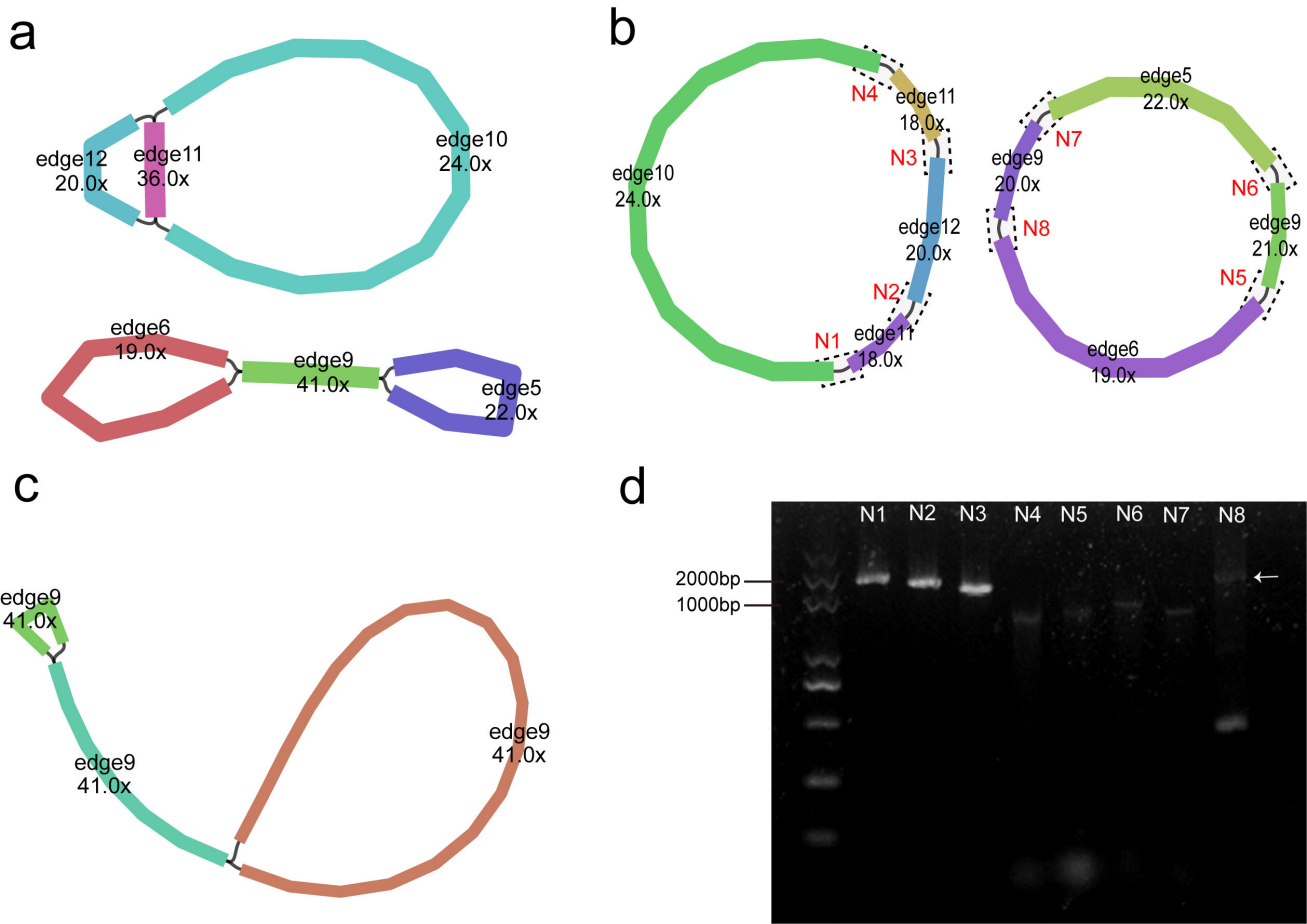
Validation of the mitochondrial genome assembly. **(a)** Initial assembly graph of the *P. edulis* mitogenome generated by oatk and himt using PacBio HiFi long reads, nodes represent contig junctions and edges correspond to assembled contigs. **(b)** Optimized conformational map of the *P. edulis* mitogenome, six contigs are connected via eight junction nodes (N1–N8), forming two major circular structures. **(c)** conformational map of the chloroplast genome; **(d)** PCR validation of the eight node regions. Agarose gel electrophoresis confirms the amplicons corresponding to N1–N8.

After sequencing and reads filtering, approximately 20 GB of NGS reads and 10 GB of TGS reads were generated. The ‘GH- 1’ mitochondrial genome exhibited a complex conformation due to the presence of a large number of repeat sequences. The PacBio HiFi reads were assembled into two “∞”−shaped conformations using oatk and himt (**Figure 1a**, contig N50 = 145,654bp). Subsequently, a hybrid assembly strategy was adopted, and the mitochondrial genome was provisionally represented as two circular molecules comprising six contigs (eight edges and eight nodes; see **Figure 1b**). The sequencing coverage depth of the mitogenome was 20× for long reads and 410× for short reads. In addition, the assembled complete chloroplast genome exhibited a typical quadripartite structure (**Figure 1c**). To further validate the connectivity of the six contigs of mitogenome, Sanger sequencing was performed using primers specifically designed for the eight node regions (node N1 to N8; primer sequences are listed in **Supplementary Table 1**). The validation results confirmed that the six contigs were joined via the eight nodes into two complete circular mitogenomes (**Figure 1d**).

### 3.2 Genome features and functional annotation of the mitogenome

**Figure 2:**
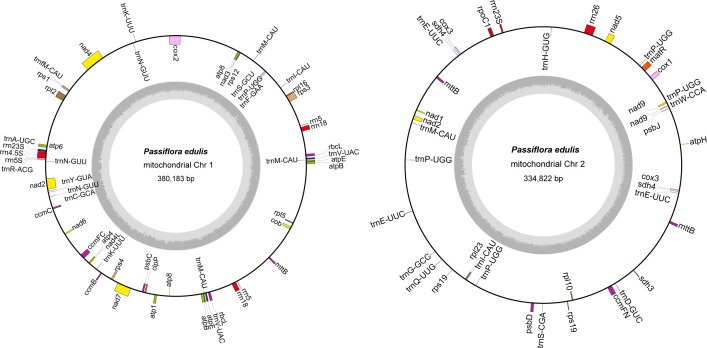
Circular map of the complete *P. edulis* mitogenome. The two circular chromosomes, mtChr1 and mtChr2, are displayed. Genes located on the outer track are transcribed clockwise, and those on the inner track are transcribed counterclockwise. Functional categories are indicated by color: Complex I in orange, Complex II in yellow, Complex III in light green, Complex IV in dark green, Complex V in blue, cytochrome genes in purple, ribosomal proteins in brown, maturase in pink, tRNAs in red, and rRNAs in gray. The inner rings show GC content (black line) and GC skew (purple and green).

The complete sequence of the ‘GH-1’ mitogenome was obtained using a hybrid assembly strategy and was verified to be a double−circular structure with a total length of 715,005 bp. Specifically, mtChr1 was 380,183 bp and mtChr2 was 334,822 bp. Both mitogenomes exhibited typical features of plant mitogenomes (**Figure 2**). GC content analysis showed that mtChr1 and mtChr2 had GC contents of 44.2% and 43.8%, respectively, which are consistent with the GC content range (42%–46%) reported for most angiosperm mitogenomes.

Gene annotation revealed that the ‘GH-1’ mitogenome of *P. edulis* encodes 50 functional genes and 21 tRNAs. These genes include core energy metabolism−related genes of NADH dehydrogenase (Complex I), succinate dehydrogenase (Complex II), cytochrome c reductase (Complex III), cytochrome c oxidase (Complex IV), and ATP synthase, as well as ribosomal protein (SSU and LSU), maturases, transfer RNA (tRNA) genes, and ribosomal RNA (rRNA) genes. Both mtChr1 and mtChr2 each contain a complete set of tRNA genes and rRNA genes (*rrn18*, *rrn26*, *rrn5*), ensuring the independence of mitochondrial gene expression (**Table 1**).

**Table 1:** Gene content and organization of the *P. edulis* ‘GH−1’ mitochondrial genome.

	Group of genes	Name of genes
core genes	ATP synthase	atp1、atp4、atp6、atp8、atpB、atpE、atpH
	cytochrome c maturation	ccmB、ccmC、ccmFC、ccmFN
	protease	clpP
	Cytochrome	cob
	Cytochrome c oxidase	cox1、cox2、cox3
	Maturases	matR
	Transport membrane protein	mttB
	NADH dehydrogenase	nad1、nad2、nad3、nad4、nad4L、nad5、nad6、nad7、nad9
Variable genes	photosystem	psbC、psbD、psbJ
	Rubisco	rbcL
	Large subunit of ribosome	rpl10、rpl16、rpl2、rpl23、rpl5
	RNA polymerase	rpoC1
	Small subunit of ribosome	rps1、rps12、rps19、rps3、rps4
	Ribosome RNA	rrn18、rrn23S、rrn26、rrn4.5S、rrn5、rrn5S
	Succinate dehydrogenase	sdh3、sdh4
	Transfer RNA	trnE-UUC、trnK-UUU、trnM-CAU、trnP-UGG、trnS-CGA、trnC-GCA、trnD-GUC、trnF-GAA、trnG-GCC、trnH-GUG、trnI-CAU、trnN-GUU、trnQ-UUG、trnR-ACG、trnS-GCU、trnW-CCA、trnY-GUA、trnA-UGC、trnV-UAC、trnfM-CAU

### 3.3 Characteristics and distribution of microsatellite repeats and long repeats

**Figure 3:**
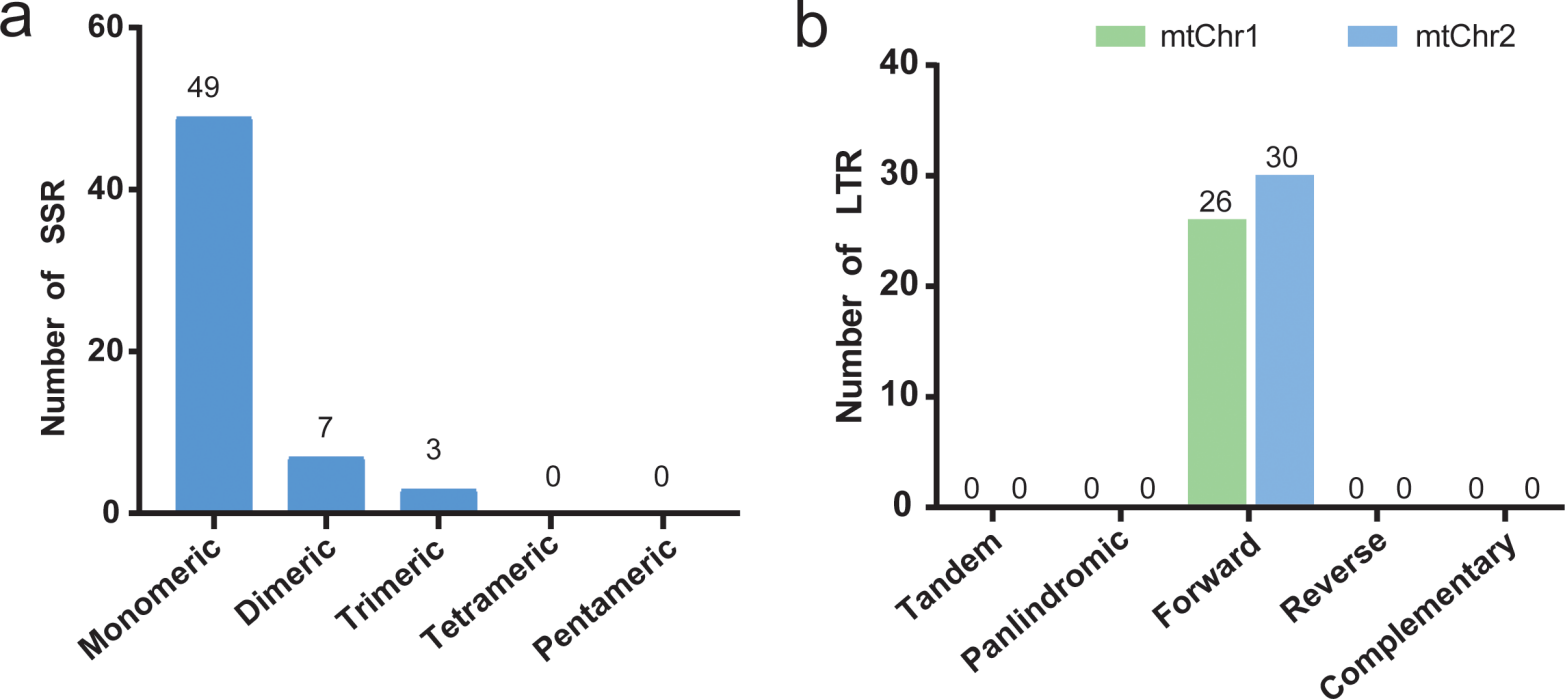
Characterization of SSRs and long repeats in the *P. edulis* mitogenome. **(a)** Frequency distribution of SSR types identified by MISA; **(b)** Length distribution of long forward repeats detected by REPuter.

A total of 59 SSR loci were identified in the ‘GH-1’ mitogenome. Among these, mononucleotide repeats were the most abundant, with 49 loci (83.05%), followed by dinucleotide repeats with 7 loci (11.86%) and trinucleotide repeats with 3 loci (5.08%). No tetra− or pentanucleotide repeats were detected (**Figure 3a**). Within the mononucleotide repeats, A/T repeats were predominant (91.84%), which is consistent with the general SSR characteristics of plant mitogenomes. SSR loci were distributed in both mtChr1 and mtChr2, with 32 loci in mtChr1 and 27 loci in mtChr2. Moreover, these SSRs were predominantly located in intergenic regions.

Long repeats analysis using REPuter software showed that only forward repeats were detected in the ‘GH-1’ mitogenome; no reverse, complement, or palindromic repeats were found. A total of 26 forward repeats were identified in mtChr1 and 30 in mtChr2, with repeat lengths mainly concentrated between 40 and 90 bp (**Figure 3b**). The longest forward repeat in mtChr1 was 30,967 bp, located in the region from 295,709 to 326,675 bp. In mtChr2, the longest forward repeat was 275 bp, located in the regions 55,533-55,807 bp and 259,968-260,242 bp, with an e−value of 8.56e−156, indicating a high degree of sequence conservation.

### 3.4 Distribution of transposable elements in the *P. edulis* mitogenome

Transposable element analysis revealed a large number of transposable elements in the *P. edulis* (‘GH1’) mitogenome. The total numbers of transposable elements were 770 in mtChr1 and 826 in mtChr2. These elements were classified into two major categories: retrotransposons and DNA transposons. Retrotransposons predominated in both circular molecules: in mtChr1, 412 retrotransposons (53.51%) and 358 DNA transposons (46.49%) were identified; in mtChr2, 459 retrotransposons (55.57%) and 367 DNA transposons (44.43%) were identified. Subclassification of retrotransposons showed that LTR (long terminal repeat) elements constituted the major component, accounting for 79.12% of retrotransposons in mtChr1 and 81.05% in mtChr2, whereas non−LTR elements represented a relatively minor fraction. This distribution pattern is consistent with the transposable element landscape reported for most land plant mitogenomes.

### 3.5 Mitochondrial-plastid horizontal gene transfer analysis

**Figure 4:**
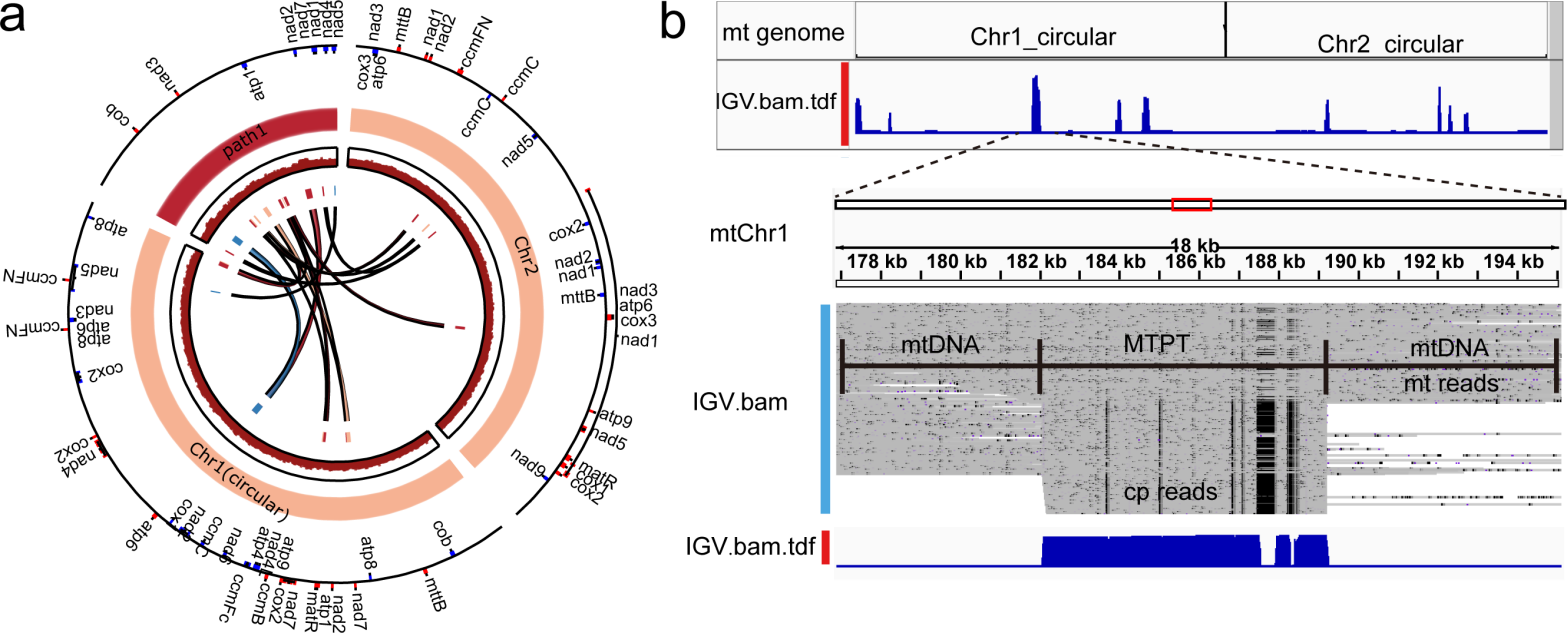
Identification of mitochondrial plastid DNA (MTPT) fragments in *P. edulis*. **(a)** Circos plot showing the genomic locations of nine MTPT fragments (MTPT1–MTPT9) identified via BLASTn (E−value ≤ 1e^−5^) using the *P. edulis* chloroplast genome as a query; **(b)** IGV screenshots of representative MTPT regions, showing coverage tracks from both PacBio HiFi long reads (upper panel) and Illumina short reads (lower panel).

Using the chloroplast genome as a reference, a BLASTn search identified nine mitochondrial plastid sequences (MTPT1 ~ MTPT9, **Figures 4a, b**) within the mitogenome. These MTPTs were predominantly located in intergenic regions, with some fragments containing chloroplast gene sequences. The MTPTs ranged in length from 1276 bp to 18,026 bp, with a total cumulative length of 43,909 bp, accounting for 6.14% of the total mitogenome length. These MTPTs contain a total of 13 chloroplast-derived mitochondrial genes or gene fragments including 5 transfer RNAs, 4 ribosomal subunits/ribosomal RNA fragments, 1 ATP synthase, 1 cytochrome c oxidase and 2 NADH dehydrogenase (**Supplementary Table 2**). The longest MTPT fragment, which included a portion of the *chloroplast cox1* gene, was 4,001 bp and was located in the region 268,776 ~ 272,776 bp of mtChr1 (**Figure 4a**). In addition, three homologous sequences of chloroplast tRNA genes (*trnM-CAU*) were detected integrated into the mitogenome, indicating that gene transfer events between the chloroplast and mitochondrion have occurred during the evolution of *Passiflora*.

### 3.6 Comparison of genomic features and gene rearrangement

**Figure 5:**
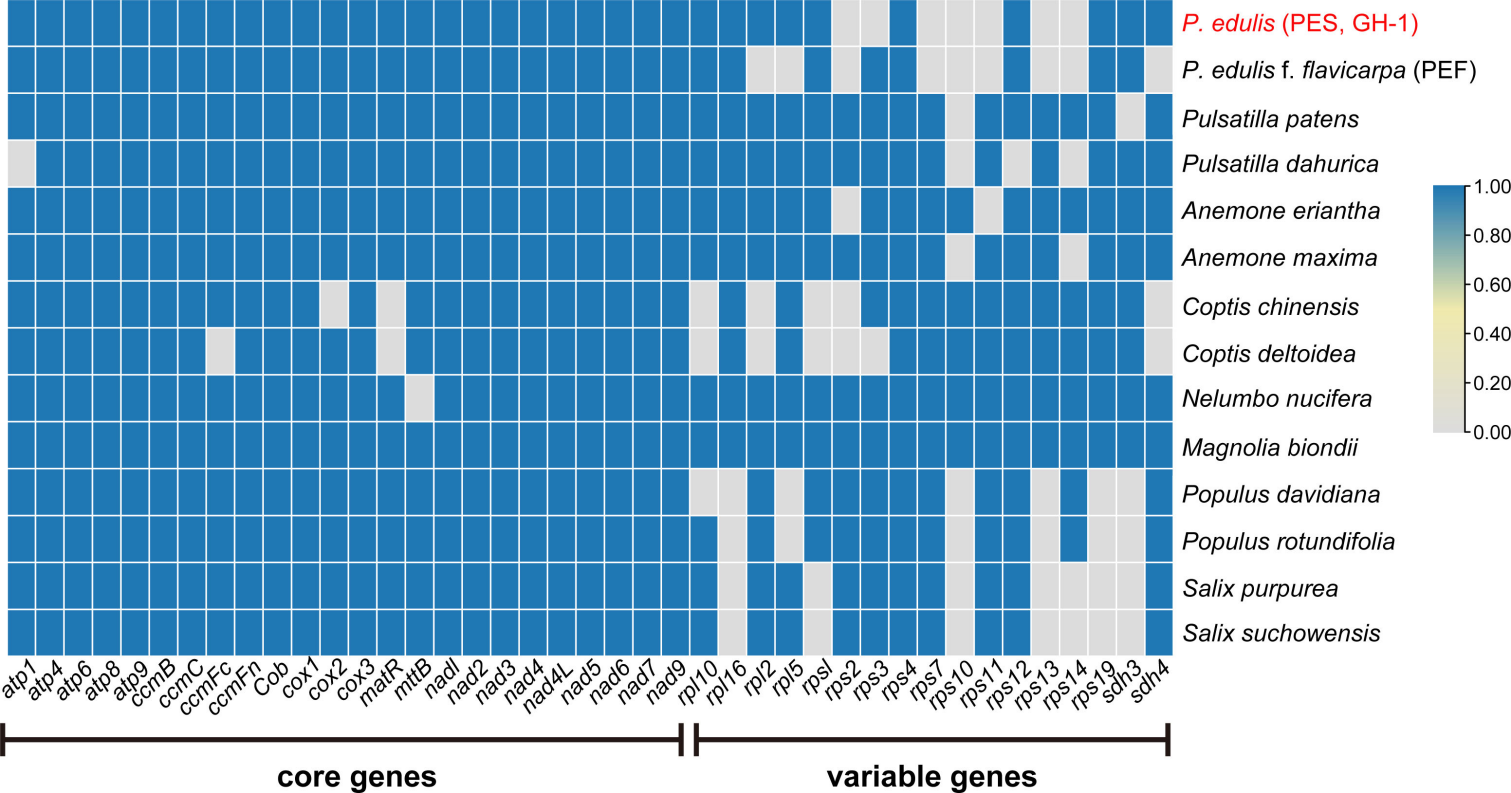
Cross−species conservation of mitochondrial protein−coding genes. Presence/absence matrix depicting the distribution of orthologous protein−coding genes across *P. edulis* and six representative angiosperms. “Core genes” are defined as those present in all analyzed species, while “variable genes” exhibit lineage−specific losses.

Comparative analysis of gene content between the ‘GH-1’ mitogenome and those of other angiosperm species revealed a conserved set of core mitochondrial genes. The ‘GH-1’ mitogenome retains 20 core protein-coding genes typically considered essential for mitochondrial function, including genes involved in oxidative phosphorylation (Complexes I-V), cytochrome c biogenesis (*ccm* genes), and ribosomal proteins (*rpl* and *rps* families)(**Figure 5**). These core genes are universally present across angiosperms, reflecting their critical roles in maintaining respiratory chain function and mitochondrial translation. However, notable variation was observed in the variable gene repertoire. Seven ribosomal small subunit genes, *i.e. rps2*, *rps3*, *rps7*, *rps10*, *rps11*, *rps13*, and *rps14*, were found to be absent from the ‘GH-1’ mitogenome. This pattern of ribosomal protein gene loss is not uncommon among angiosperms and has been documented in multiple lineages. Even within the same species, PEF and PES exhibit individual differences in variable genes. For instance, the *rps2* gene was not detected in the mitogenome of PES compared with PEF, whereas PEF lacked a greater number of variable genes (*rpl2*, *rpl5*, *rps1*, and *sdh* genes).

**Figure 6:**
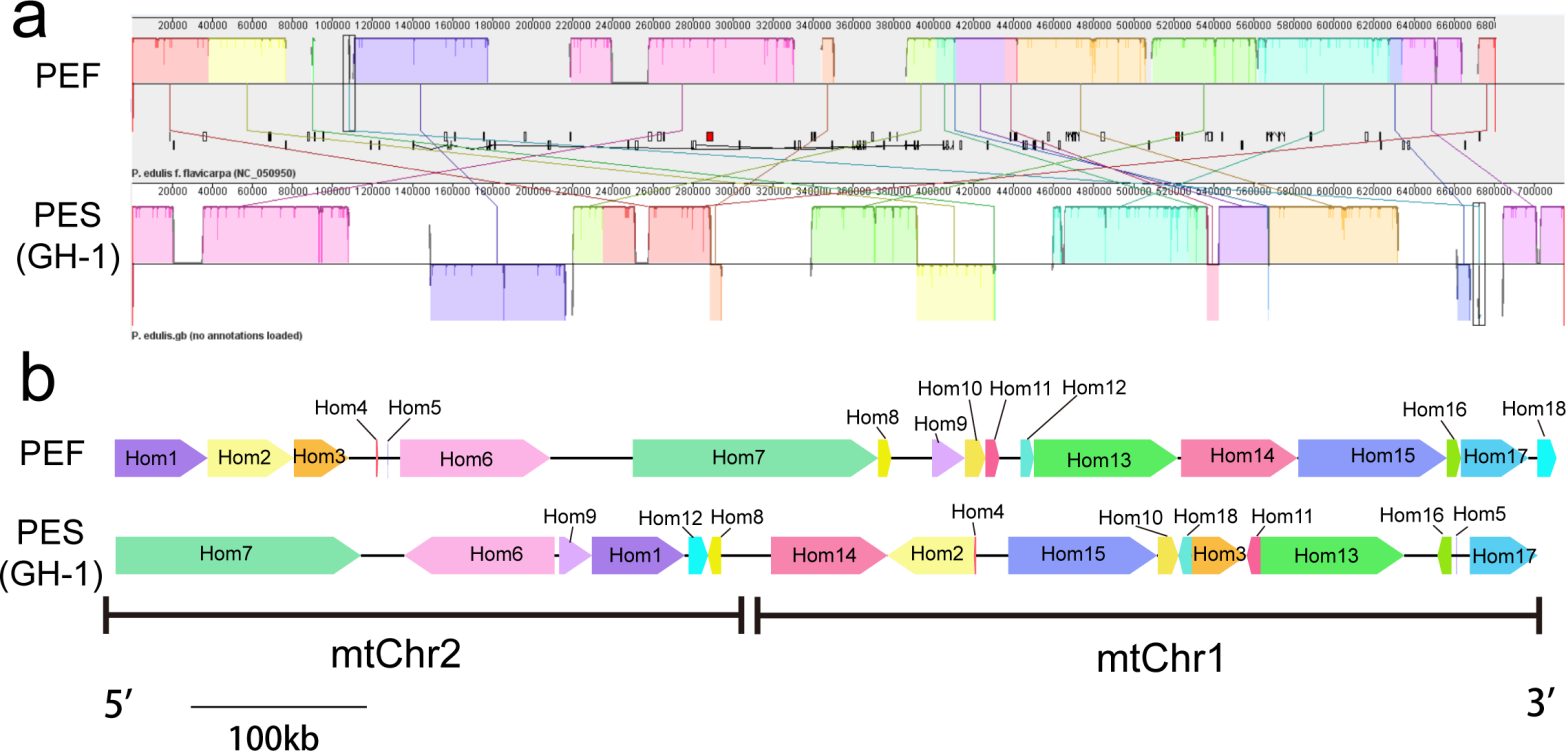
Inter−varietal synteny and gene rearrangement between purple (PES, ‘GH−1’) and yellow (PEF) *P. edulis* mitogenomes. **(a)** Conserved syntenic blocks are connected by colored ribbons, with the PES mitogenome assembly serving as the reference and PEF as the query; **(b)** detailed gene order comparison within six representative syntenic blocks. Genes are depicted as colored arrows, with arrow direction indicating transcriptional orientation. Homologous genes are linked by the same color (hom1–hom18). Extensive gene rearrangements, including inversions and translocations, are evident between the two cultivars.

To investigate the genome feature and homologous gene rearrangements of the mitochondrial genomes between the PEF and PES varieties of passion fruit, we further performed a comparative genomic analysis between the two cultivars. A total of 18 conserved regions (hom1 ~ hom18) were identified (**Figure 6a**). These conserved regions were extracted and used to detect homologous gene rearrangements. Among them, six conserved regions were located on mtChr2, and twelve on mtChr1 (**Figure 6b**). Compared with the previously reported PEF mitogenome, most of the conserved regions in the PES mitogenome exhibited gene rearrangements, but without displaying a clear pattern. Compared with the previously reported PEF mitogenome, most of the conserved regions in the PES mitogenome exhibited gene rearrangements, but without displaying a clear pattern.

### 3.7 Codon usage bias and RNA editing site analysis

**Figure 7:**
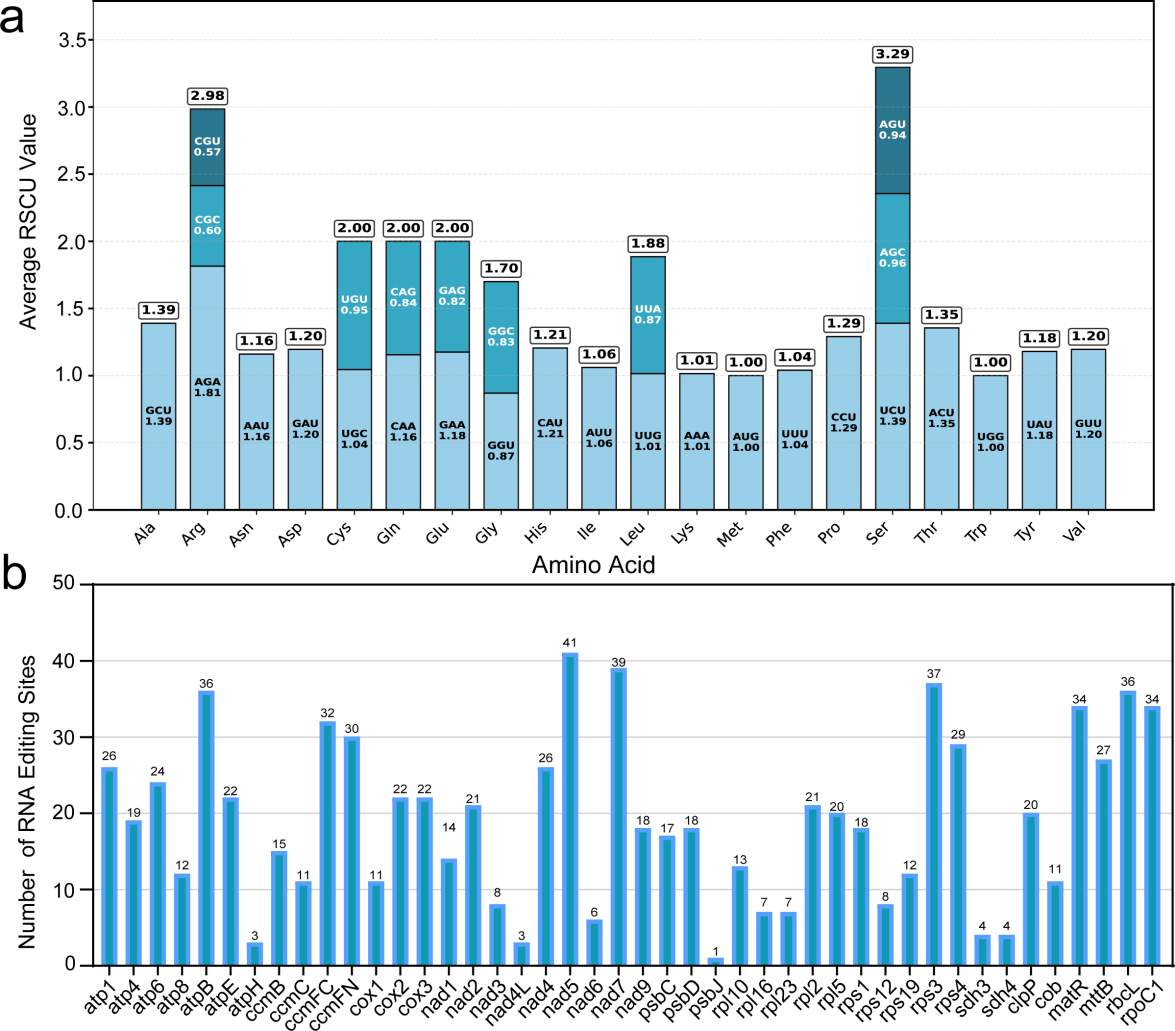
Codon usage bias and RNA editing sites in the *P. edulis* mitogenome. **(a)** Heatmap of relative synonymous codon usage **(RSCU)** values for all mitochondrial protein−coding genes. RSCU values were calculated using CodonW; **(b)** distribution of C−to−U RNA editing sites predicted by the PMGA pipeline. The left panel shows the number of editing sites per protein−coding gene. The right panel displays the proportion of editing events occurring at the first, second, and third codon positions.

Codon usage bias analysis revealed a marked preference in the protein−coding genes of the *P. edulis* mitogenome (**Figure 7a**). With the exception of the start codon AUG and the tryptophan codon UGG (which has only one synonymous codon), some codons for each amino acid exhibited RSCU values greater than 1. For example, among the codons encoding arginine (Arg), AGA (1.81) had the highest RSCU value (1.81); for serine (Ser), UCU (1.39); and for alanine (Ala), GCU (1.39) was the preferred codon. Codon usage bias is primarily associated with gene expression level, tRNA abundance, and genomic GC content.

A total of 392 RNA editing sites were predicted in the Passiflora mitogenome, covering 44 protein−coding genes (**Figure 7b**). All editing events were of the C−to−U type. The number of editing sites varied considerably among genes. The *nad5* gene contained the highest number of editing sites (41), followed by *nad7* (39), *rps3* (37) and *atpB* (36), whereas *atpH* (3), *nad4L* (3) and *psbJ* (1) had the fewest. Analysis of codon position distribution showed that editing sites were predominantly located at the second codon position (68.37%), followed by the first position (27.55%), with the third position being the least represented (4.08%). This distribution pattern is consistent with the general feature of RNA editing in plant mitogenomes, namely that editing at the second codon position is more likely to result in amino acid changes and thus affect protein function.

### 3.8 Phylogenetic analysis

**Figure 8:**
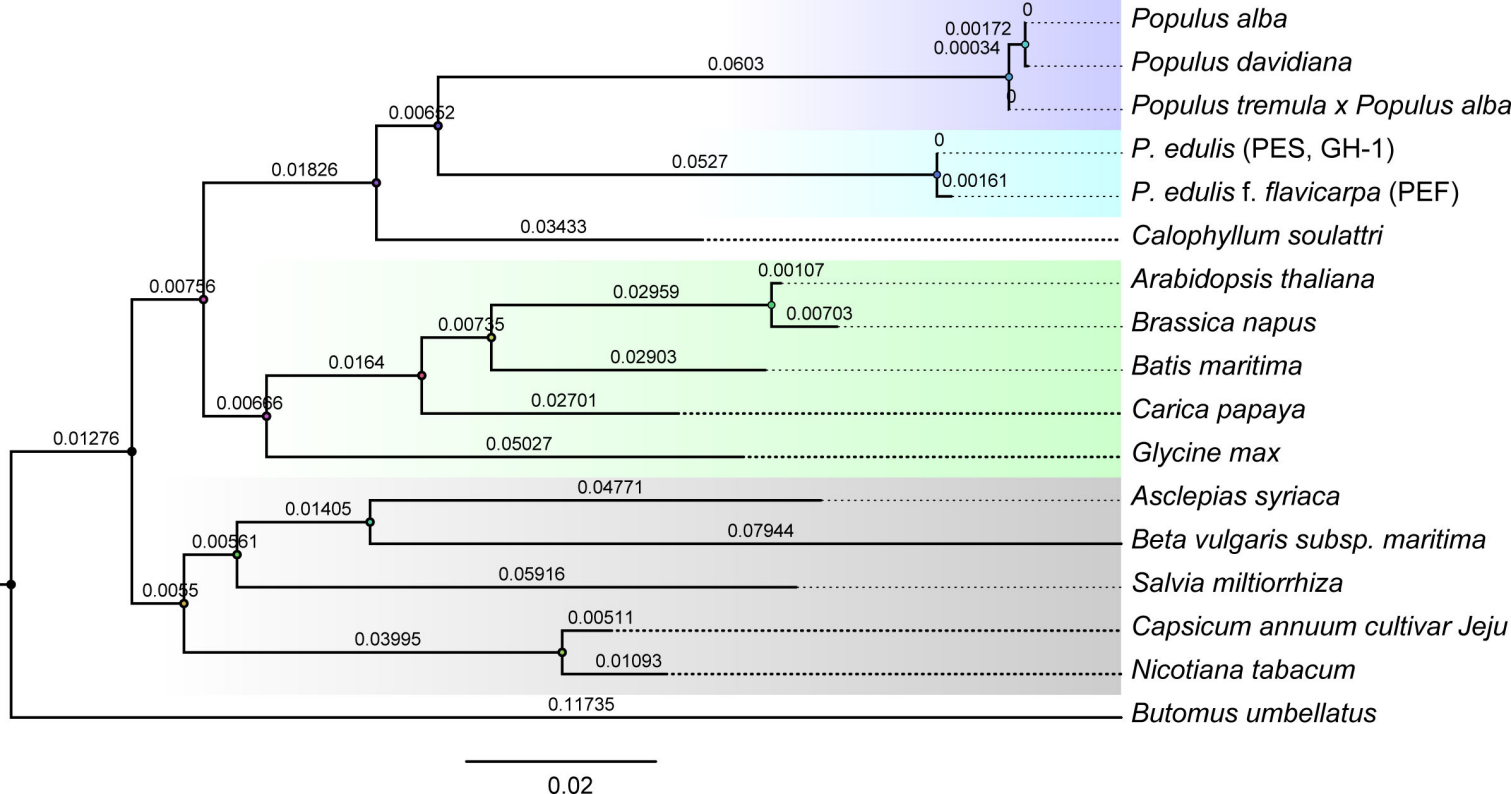
Phylogenetic inference of *P. edulis* and related angiosperm species based on mitochondrial genome sequences.

A phylogenetic tree was constructed based on shared protein−coding genes from the mitochondrial genomes of 16 representative angiosperm species. The results showed that *Passiflora* formed an independent branch with species of the same family (Passifloraceae), with a bootstrap support value of 100%, confirming its unambiguous phylogenetic position (**Figure 8**). In terms of evolutionary relationships, Passiflora exhibited relatively closer affinity with species of the Salicaceae (genus *Populus*), whereas it was more distantly related to members of the Brassicaceae (*A. thaliana*, *Brassica napus*) and Fabaceae (*Glycine max*). This pattern is largely consistent with traditional taxonomic classifications and with phylogenetic analyses based on chloroplast genome data (Cauz-Santos et al., 2017). The branching structure of the phylogenetic tree reflects the evolutionary trajectory of angiosperm mitogenomes, namely that the evolutionary rates of mitogenomes within the same family or genus tend to be relatively uniform, whereas significant genetic divergence exists among different families.

## 4 Discussion

Mitochondria provide plants with the energy required for growth, development, and stress adaptation (Atkin and Macherel, 2009; Barreto et al., 2022). Plant mitochondrial genomes exhibit remarkable structural diversity, and they are considerably more complex than their animal counterparts, largely due to their larger sizes and higher abundance of repetitive sequences (Newton, 1988). Studies based on Sanger or next−generation sequencing (NGS) often failed to resolve the true three−dimensional conformation of plant mitogenomes. Most reported plant mitogenomes were assembled as single−circular or even non−circular structures, with further manual integration into a draft circular mitogenome (Yang and Wang, 2020). Recent advances in long−read sequencing technologies and improvements in assembly algorithms have made it possible to resolve complex mitochondrial genome structures using third−generation long reads. In this study, we assembled the complete *P. edulis* mitogenome using a hybrid sequencing approach. The *P. edulis* mitochondrial genomes displayed a double−circular structure, with each circle possessing a complete set of genes and independent replication units. Compared with the previously reported draft single−circle mitogenome of *P. edulis* f. *flavicarpa* (Yang and Wang, 2020), our assembly shows substantial improvements in both assembly completeness (N50, 145,654bp) and the quantity and quality of annotated mitochondrial genes (complete core genes and rich variable genes). Therefore, this assembly can serve as a reliable reference mitogenome for future studies on *Passiflora*.

To date, high−quality complete plant mitogenomes have been found to adopt various architectures, including single−circle, multi−circle, linear, and branched configurations. For example, PacBio−based sequencing revealed that the poplar mitogenome comprises up to nine circular conformations (Qu et al., 2023), and the model plant *A. thaliana* also harbors two complete circular mitogenomes (Park et al., 2021). The formation of the double−circular conformation in *Passiflora* is likely associated with recombination events mediated by repetitive sequences. Long repeats act as recombination hotspots, promoting mitogenome rearrangement and circularization. The abundance of forward repeats identified in the *Passiflora* mitogenome may provide a molecular basis for maintaining the double−circle architecture. Whether this double−circular structure confers unique advantages in energy metabolism efficiency or gene expression regulation remains to be further investigated using transcriptomic and proteomic data.

Inter−organellar gene transfer between mitochondria and chloroplasts represents a fascinating and complex aspect of plant genome evolution (Choubey and Rajam, 2015). In seed plants, the transfer of DNA from the chloroplast to the mitochondrion is well documented and often involves tRNA genes, ribosomal RNA fragments, and protein−coding sequences (Dietrich et al., 1992). These transferred regions may become functional, pseudogenized, or further mobilized, thereby contributing to the structural plasticity and expanded size of plant mtDNA. Episodes of large−scale chloroplast−to−mitochondrion transfer have been identified in diverse lineages, including legumes, geraniums, and sweet potato, often coinciding with increased genomic rearrangements and accelerated nucleotide substitution rates (Li et al., 2024; Park et al., 2017). In *P. edulis*, 14 MTPT genes were identified, nearly one-half of them were the transfer RNA. These processes are likely mediated by recombination machinery, double−strand break repair, and possibly relaxed DNA surveillance mechanisms, ultimately generating genomes that are evolutionary composites.

Conversely, mitochondrial−to−plastid transfer appears rarer but has been reported in several groups, reinforcing the notion that intracellular DNA movement is bidirectional and ongoing (Torralba et al., 2016). Besides, extensive structural rearrangements, including inversions, expansion/contraction of the inverted repeat, and multiple gene losses were identified in *P. edulis*. These features are coupled with accelerated substitution rates in several plastid genes (e.g., *rrn5/18*, *rrnS/rrnL*, ribosomal subunits) and suggest a syndrome of genomic instability analogous to that found in Passiflora.

This study represents the comprehensive analysis of the mitochondrial genome in *Passiflora*, providing critical insights into the evolutionary dynamics of organellar genomes in this economically important genus. By employing advanced sequencing and bioinformatic approaches, we have assembled a high-quality mitochondrial genome (715,005bp divided into two circular) enabling detailed characterization of gene content, organization, and evolutionary patterns. Notably, even within the same species of *P. edulis*, PEF and PES exhibit individual differences, whereas PEF lacked a greater number of variable genes (*rpl2*, *rpl5*, *rps1*, and *sdh* genes). Our findings reveal the unique structural features of ‘GH-1’ mitochondrial genomes, including extensive repeat sequences and transposable elements, and provide the first evidence of horizontal gene transfer between chloroplast and mitochondrial genomes in *Passiflora*. The identification of gene transfer events and genome rearrangements in *Passiflora* will advance our understanding of organellar genome evolution as well as provide critical insights for phylogenetic studies, hybrid identification, and breeding programs within this economically important genus.

## 5 Conclusion

This study presents the complete assembly of the *P. edulis* mitogenome, revealing a double-circular architecture (380,183 bp and 334,822 bp) rather than the previously assumed single circle. We identified 59 SSRs, 56 long forward repeats, and 1,596 TEs, predominantly LTR retrotransposons. Extensive mitochondrial plastid DNA transfers (MTPTs) were quantified, accounting for 6.14% of the mitogenome and including 13 chloroplast-derived gene fragments. Comparative analysis between purple (‘GH-1’, PES) and yellow passion fruit (PEF) uncovered substantial gene rearrangements and lineage-specific variable gene losses, highlighting dynamic mitogenome evolution even within the same species. These findings provide a high-quality reference for Passifloraceae genomics and underscore the role of inter-organellar gene transfer and repetitive elements in shaping structural complexity, laying a foundation for future functional and breeding studies in passion fruit.

## Data Availability

The datasets presented in this study can be found in online repositories. The names of the repository/repositories and accession number(s) can be found below: https://db.cngb.org/, CNP0009297, CNS1506227, CNA0703064, CNA0703065.

## Supplementary material

Supplementary Table 1 Primer information in this study.

Supplementary Table 2 MTPTs between chloroplast and mitochondrial in *P. edulis*.
